# Zebrafish models of sarcopenia

**DOI:** 10.1242/dmm.042689

**Published:** 2020-03-30

**Authors:** Alon Daya, Rajashekar Donaka, David Karasik

**Affiliations:** 1 The Faculty of Marine Sciences, Ruppin Academic Center, Michmoret 40297, Israel; 2 The Musculoskeletal Genetics Laboratory, The Azrieli Faculty of Medicine, Bar-Ilan University, Safed 130010, Israel; 3 Hebrew SeniorLife, Hinda and Arthur Marcus Institute for Aging Research, Boston, MA 02131, USA

**Keywords:** Zebrafish, Sarcopenia, Aging, Genome, GWAS, Muscle disease, Imaging

## Abstract

Sarcopenia – the accelerated age-related loss of muscle mass and function – is an under-diagnosed condition, and is central to deteriorating mobility, disability and frailty in older age. There is a lack of treatment options for older adults at risk of sarcopenia. Although sarcopenia's pathogenesis is multifactorial, its major phenotypes – muscle mass and muscle strength – are highly heritable. Several genome-wide association studies of muscle-related traits were published recently, providing dozens of candidate genes, many with unknown function. Therefore, animal models are required not only to identify causal mechanisms, but also to clarify the underlying biology and translate this knowledge into new interventions. Over the past several decades, small teleost fishes had emerged as powerful systems for modeling the genetics of human diseases. Owing to their amenability to rapid genetic intervention and the large number of conserved genetic and physiological features, small teleosts – such as zebrafish, medaka and killifish – have become indispensable for skeletal muscle genomic studies. The goal of this Review is to summarize evidence supporting the utility of small fish models for accelerating our understanding of human skeletal muscle in health and disease. We do this by providing a basic foundation of the (zebra)fish skeletal muscle morphology and physiology, and evidence of muscle-related gene homology. We also outline challenges in interpreting zebrafish mutant phenotypes and in translating them to human disease. Finally, we conclude with recommendations on future directions to leverage the large body of tools developed in small fish for the needs of genomic exploration in sarcopenia.

## Abstract (precis)

**Summary:** Zebrafish and other small fish have become powerful disease models. Here, we summarize the evidence for the utility of small teleost models for genetic research in sarcopenia – the age-related loss of muscle mass and function.

## Introduction

Muscle health is an essential element of healthy life and successful aging. Skeletal muscle represents the largest organ system, comprising 35-50% of the body mass (Brotto, 2018). Sarcopenia, or loss of muscle mass, and dynapenia (see Glossary, Box 1), the loss of muscle strength, pose significant problems for the aged, especially as life expectancy rises in developed countries (Froehlich et al., 2013). Reductions in muscle mass, strength and physical function are virtually universal with aging. They are associated with falls, disability, institutionalization and mortality, resulting in a considerable impact on the rapidly growing aged population and a substantial bearing on health care expenditures. Few interventions, if any, have been successfully tested to prevent or treat sarcopenia, dynapenia and mobility limitation (Dent et al., 2018). The task force of the International Conference on Sarcopenia and Frailty Research (ICSFR) recommended resistance-based training, protein supplementation and/or a protein-rich diet, and an appropriate caloric intake rather than pharmacological interventions (Dent et al., 2018).

Notably, the degree of muscle loss and decline in physical performance in older people is highly variable and seems to be regulated by intrinsic factors. Muscle loss etiology is likely to be due to tissue-level molecular perturbations in key biological pathways. Understanding these perturbations is essential for developing efficient approaches to more effectively detect, prevent and treat the loss of muscle mass and performance.

### Diagnosis of sarcopenia

Traditionally, muscle mass and muscle strength (force) were key indicators for a diagnosis of sarcopenia. Recently, metrics of poor physical functioning were added for defining sarcopenia (Cruz-Jentoft et al., 2019). A sarcopenia-related concept, frailty syndrome, is specifically defined by low grip strength, low walking speed and low physical activity (Cesari et al., 2006).

With all the progress in the disease definition, its clear framing and operationalization remain challenging; only recently (2016) was sarcopenia recognized as a nosologic entity (Rolland, 2016; Cawthon et al., 2019). Furthermore, the diagnostic algorithms used to define the sarcopenic individual attempted to consider the measured components of physical function and muscle strength differently (Cruz-Jentoft et al., 2014; Fielding et al., 2011). Some argued that the predictive value of sarcopenia definitions for negative health-related events is largely driven by the skeletal muscle ‘quality’ (physical performance measures) rather than its ‘quantity’ (skeletal muscle mass) (Rolland, 2016). Others argued that the term sarcopenia considers primarily muscle's bulk, whereas the loss of muscle strength, dynapenia, is more important for the disease outcomes and thus diagnosis. The two dimensions of sarcopenia are still of equal weight in clinical diagnosis (Rolland, 2016), but this balance might change (Cawthon et al., 2019).

Although also involving atrophy, seen particularly at the cellular level, cachexia (Box 1) usually develops secondary to some damaging factors or catabolic disease. Cachexia seems to share some etiology with idiopathic sarcopenia, while remaining a separate entity, although genetic predisposition to cancer cachexia seems to overlap with that for sarcopenia (Byrne et al., 2019).

There are also debates as to whether sarcopenia is a reflection of normal aging or is indeed a stand-alone nosologic entity. Importantly, from the point of view of genomic exploration, there is no difference; the ability to accurately phenotype larger human cohorts is a key for success of genomic studies. When studying sarcopenia, both muscle mass and muscle strength are measurable exophenotypes (Box 1), while endophenotype (Box 1) measures – those more biologically proximal to the gene actions – are currently lacking. Researchers will need these endophenotypes to approximate physiology from a gene's function. Examples from other fields include blood levels of C-reactive protein for pro-inflammatory conditions or of creatinine for kidney functions. Muscle exo- and endophenotypes can be analyzed either as a continuous (lean mass, grip strength) or dichotomous (diagnosis of sarcopenia or dynapenia, yes/no) trait in genotype-phenotype association studies.

Given that the cellular and biochemical factors (the aforementioned endophenotypes) affecting muscle structure and composition in aging are still uncertain, measuring or imaging body composition offers a rough approximation. The two standard techniques available for body composition parameter quantification are dual X-ray absorptiometry (DXA), which estimates fat-free (lean) and fat mass (Engelke et al., 2018), and bioelectrical impedance (Kyle et al., 2003). Both bioelectrical impedance and DXA cannot provide a spatially resolved distribution of muscle or adipose tissue. This is the domain of computed tomography (CT) and magnetic resonance (MRI) techniques (Engelke et al., 2018). However, the spatial resolution of CT and MRI still might not be informative, especially in elderly and diseased individuals with a high and inhomogeneous muscle fat ‘infiltration’ (Engelke et al., 2018).

Fatty infiltration and replacement of fast with slow muscle fiber types, together with fibrosis, are probably the most obvious histological phenotypes one needs to consider to define muscle pathology. Yet, objective methods such as biopsy are invasive and thus problematic in large samples of humans, especially those unaffected by muscle loss, and the histological and immunohistochemical parameters for sarcopenia are not standardized (Trovato et al., 2016). Also, given that hundreds of muscles exist and behave as (semi-) independent organs, the choice of correct site for biopsy is currently arbitrary. This overall lack of standardization further complicates the accuracy of a diagnosis of sarcopenia.

Several molecular pathways have been suggested to accelerate muscle mass loss; however, to date, these have not translated to useful circulating, imaging or epigenetic biomarkers (Box 1) to identify persons at risk of sarcopenia early enough before structural changes in skeletal muscle progress to the point of impairing function (Cruz-Jentoft et al., 2019). A novel approach, based on the dilution of an oral dose of creatinine-(methyl-deuterium, D_3_) [termed D_3_-Cr dilution] determined by D_3_-Cr enrichment in urine, is receiving increasing attention for its potential application to quantify muscle mass (Evans et al., 2019). Unfortunately, the detection of D_3_-Cr requires sophisticated spectrometry, which limits this method to well-equipped medical and research centers. In addition, the D_3_-Cr technique only provides estimates of total muscle mass with no information on muscle function. Therefore, a method that measures the capacity for mitochondrial energy production by calculating the maximum mitochondrial adenosine triphosphate (ATP) production (ATPmax) *in vivo* was proposed. It is based on the idea that diminished mitochondrial function may contribute to higher levels of fatigability in older adults (Santanasto et al., 2015). Another potential marker of muscle energy metabolism, phosphocreatine recovery, is measured using ^31^P magnetic resonance spectroscopy. However, larger cohort studies using this marker are again compromised by the sophisticated quantification technique that demands precise machinery. Therefore, large-scale genetic studies of these important endophenotypes are not possible at present, and researchers are currently focusing on exophenotypes.

**Box 1. Glossary**

**Biomarker:** characteristic that can be objectively measured to capture a biological or pathogenic process, or response to pharmacologic intervention.

**Cachexia:** muscle volume loss, usually as a result of catabolic disease.

**Dynapenia:** loss of muscle strength with aging.

**Endophenotype:** a heritable biological marker that can bridge the gap between high-level symptom presentation and low-level genetic variability.

**Exophenotype:** measurable, obvious and external symptom or trait (i.e. behavior or physical appearance).

**Fast muscle:** also referred to as white muscle. Muscle fibers for which functions are fueled by glycolytic metabolism.

**Linkage disequilibrium:** non-random association of polymorphic alleles at different loci in a given population, whereby haplotypes do not occur at the expected frequencies.

**Muscular dystrophy:** a muscle weakness caused by the breakdown of muscle fibers.

**Myopathy:** lack of essential muscle developmental components or a delay of muscle repair after damage.

**Myotome:** a hub for muscle fibers, controlled by a single nerve root.

**Ohnologs:** gene duplicates originating from whole genome duplication (as distinguished from orthologs).

**Orthologs:** genes in different species descending from a common ancestor and encoding proteins with the same function.

**Slow muscle:** also referred to as red muscle. Muscle fibers for which functions are fueled by oxidative metabolism.

## Uncovering the genomic architecture of sarcopenia

Exophenotypes measuring various facets of sarcopenia have a strong genetic component, with heritability estimates above 50% (Willems et al., 2017). The heritability of lean muscle mass was estimated at 42% (Karasik et al., 2009) and that of grip strength ranged between 40% and 65% (Matteini et al., 2016). Having such a substantial heritability makes a genetic exploration of sarcopenia a promising undertaking. Of note, observing changes in the trait is usually quite a different phenotype from the ‘snapshot’ one-time measure of that trait. Therefore, given the lack of prospective biomarkers that would allow researchers to track changes in lean muscle mass over a long period of time, and the above-discussed limitations of the measuring techniques for the existing candidate biomarkers, the current studies on the heritability of changes in lean mass or muscle strength do not yet provide complete answers.

As soon as heritability is ascertained, genome-wide association studies (GWAS) of sarcopenia become justified. In GWAS, millions of variants [usually single nucleotide polymorphisms (SNPs)] are genotyped in thousands of individuals who are also measured for muscle phenotypes. For each SNP, a test is performed to determine whether a phenotype (dichotomous or quantitative) is different in individuals carrying different variants. Large-scale GWAS consortia continue to discover muscle-related loci through amassing data from immense cohorts of consistently phenotyped people. For example, international consortia have published the largest GWAS meta-analyses of lean mass (Zillikens et al., 2017; Karasik et al., 2018) and hand grip strength (Matteini et al., 2016; Willems et al., 2017; Tikkanen et al., 2018) to date.

SNPs in close genomic proximity tend to be genetically linked, a phenomenon called linkage disequilibrium (Box 1). Thus, SNPs with significant associations usually cluster within chromosomal loci. A usual concern in GWAS is that ∼90% of disease-associated variants reside in non-coding regions (Maurano et al., 2012). These polymorphisms most probably have regulatory functions, sometimes affecting not only their flanking GWAS-discovered loci, but also distant genes. To adequately interpret these complex interactions, researchers need a thorough understanding of the functional relationships within the coding and regulatory genome.

### Role of GWAS in discovery of novel pathways and underlying biology

The structure and function of skeletal muscles can be regulated by multiple mechanisms, including anatomical (anabolic/catabolic, vascular), physiological (hormonal milieu, satiety) or even behavioral (desire to exercise, pain tolerance). There are known molecular pathways that contribute to muscle mass, including the growth hormone/IGF-1 axis, WNT signaling, sex steroids and the TNF-α (also known as TNF)/NF-κB (also known as NFKB1) pathway (reviewed in Karasik and Kiel, 2010; Brotto and Abreu, 2012 and most recently in Brotto, 2018).

The list of genetic pathways responsible for regulating muscle-related traits is still far from complete. Although GWAS have revolutionized our understanding of the genetic architecture of multiple complex traits and diseases, they are also entering the field of sarcopenia. Of note, leg strength (Matteini et al., 2016), walking (gait) speed (Ben-Avraham et al., 2017) or frailty (Sathyan et al., 2018) GWAS could not identify statistically significant genome associations, probably due to small sample sizes and/or phenotype measurement uncertainty, which decreased the signal-to-noise ratio.

Conversely, two recent studies found statistically significant associations, with the first identifying seven loci (named by their proximal gene as *HSD17B11*, *VCAN*, *ADAMTSL3*, *IRS1*, *TNRC6B*, *MC4R* and *FTO*) robustly associated with either total and/or appendicular lean mass (Karasik et al., 2018), which constitutes a good proxy for skeletal muscle mass. The second GWAS focused on grip strength and used the large UK Biobank sample, yielding 64 muscle-strength-related loci (Tikkanen et al., 2018). Interestingly, although focusing on complex exophenotypes, these GWAS identified some genes previously recognized for rare and Mendelian diseases, such as *KIF1B*, a candidate for Charcot-Marie-Tooth disease type 2A; *MC4R*, which is implicated in autosomal-dominant obesity; and *FTO*, which is associated with congenital anomalies, further highlighting the need for accurate functional annotation of sarcopenia-related genes.

### Systematic analysis of gene function

Functional annotation of the majority of candidate sarcopenia-related genes will form the basis for a mechanistic understanding of loci potentially influencing muscle, and, in turn, help translate these candidates into clinical targets. It is important to realize that drug targets supported by genetic evidence such as GWAS are two to three times more likely to pass through a pharmaceutical pipeline of clinical development (Nelson et al., 2015). To sum up, due to the resolution of GWAS, the causative gene at a particular locus often remains unidentified, as many genes can lie within the same genetic interval. Thus, the power in this genetic approach is bound to remain unharnessed without extensive, and often cost-prohibitive, laboratory tests, including animal testing. However, the systematic functional assessment of a large number of potential candidates in a rodent model will not be efficient or cost-effective. At the same time, a growing number of studies suggest that homology between small fish and mammals is deeply conserved (see Witten et al., 2017), making them a model of interest.

## Fish as models for sarcopenia research

Aging-related conditions, such as kyphosis and muscle weakness, are observed in small fish (Kishi et al., 2003). Species such as the guppy, red panchax, medaka, platyfish and perch exhibit gradual senescence, which is typical for most vertebrates. Additionally, the African turquoise killifish is an example of an emerging experimental model for aging, including musculoskeletal detriment (Kim et al., 2016; Li et al., 2020). Indeed, despite their short lifespan compared to other vertebrates, various strains of the killifish manifest senescence, including decline in reproduction and fertility, cognition, mobility, regeneration and tissue homeostasis, along with increased incidence of neural and muscular degeneration, including sarcopenia, and cancerous lesions (reviewed in Hu and Brunet, 2018). Older fish are paler with loss of color patterns on fins, exhibit tissue homeostasis defects, fail to properly heal wounds, display spine bending (kyphosis), exhibit muscle loss (e.g. in the dorsal region) and have difficulty swimming properly (Hu and Brunet, 2018). Thus, these fish recapitulate numerous stereotypical aging traits that have been reported in other vertebrates, therefore supporting the use of small fish as models for diseases of human aging.

**Fig. 1.**
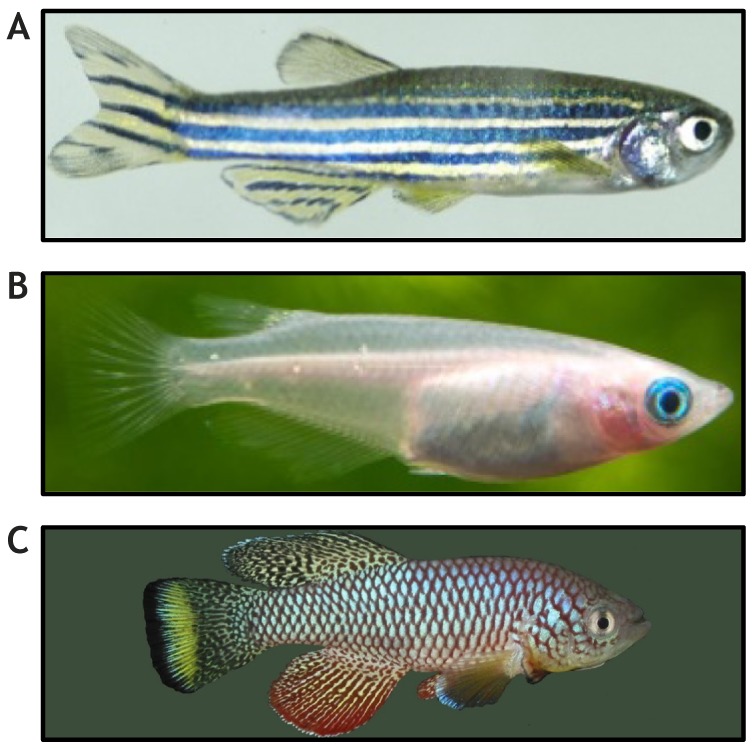
**Small teleost fish that can be used to model the genomics of sarcopenia.** (A) Zebrafish. Image from http://www.fishtankmaintenance.net/zebra-fish-zebrafish-facts/. (B) Medaka. Image from https://aquaticarts.com/collections/killifish/products/pearl-medaka-ricefish. (C) Killifish. Image from https://confessionsofafishgeek.blogspot.com/2013/02/background-nothobranchiusfurzeri-is.html. These images are not published under the terms of the CC-BY license of this article. Please refer to individual sites for further details.

Several teleost fishes, such as tilapia, trout or carp, are also important for commercial fisheries and industrial aquaculture. In these economically important fishes, the muscle components that can be modulated by genetics or environmental factors are crucial to achieve production efficiency and improve meat/flesh quality. Such research is allowing cross-fertilization between the human- and animal-genetics fields (Phelps et al., 2013; Schartl, 2014), and could provide important insight into human skeletal muscle disorders, such as sarcopenia. Although research in medaka (*Oryzias latipes*; Fig. 1B) and killifish (*Nothobranchius furzeri*; Fig. 1C) is providing valuable insight, the zebrafish (*Danio rerio*; Fig. 1A) remains the most commonly used teleost model species, also because its genome was sequenced early on. Therefore, the rest of this Review will focus on the prospects and challenges of systematic muscle-related gene function research in zebrafish.

### Zebrafish

Zebrafish are a small-bodied tropical freshwater fish species from South Asia. The ease of care, year-round prolific breeding, transparent eggs and larvae, and external development have made these fish a popular vertebrate model for developmental biology research and insight into many diseases. Over the past 40 years, the advancement of methods for genetic and developmental manipulation had driven a rapid rise in their use. Specifically, its adoption as a key genetic model for vertebrate genetics – beyond development – had become possible thanks to site-directed mutagenesis approaches to target the critical domains affected by human disease-causing mutations.

Although animal models usually struggle to recapitulate all features of human disease, zebrafish have a number of significant advantages for muscle research. Skeletal muscle makes up a considerable portion of the zebrafish trunk and has an exceptionally high degree of similarity to human muscle both molecularly and histologically. The ease of breeding and maintenance allows researchers to quantify multiple phenotypic traits in large populations through time, therefore allowing studies of genetically similar animals, such as siblings and half-siblings.

Researchers can easily and non-invasively assess the disruption of muscle structure via e.g. a birefringence assay, which examines changes in the polarity of light as it travels through the highly ordered muscle structure. Zebrafish can also readily absorb drugs added to their water and have been successfully utilized for large-scale drug screens to rescue damage to muscles (Baxter and Bryson-Richardson, 2018). Moreover, zebrafish was proposed as a model for the study of aging over a decade ago (Kishi et al., 2003). Markers and hallmarks of ageing that were demonstrated in zebrafish include oxidized protein accumulation in muscle, age-dependent mitochondrial dysfunction, telomere deterioration, spinal curvature (kyphosis), senescence-associated beta-galactosidase activity in skin, decline in regenerative capability and increased accumulation of lipofuscin (aging pigment) in the liver. Muscle function follows a decline in whole-organism performance and trainability with age. Similar to humans, exercise training improves swimming performance in young (8-12 months) and middle-aged (15-20 months) zebrafish, but not in old (25-30 months) zebrafish (Gilbert et al., 2014).

## Comparative skeletal muscle anatomy, development and growth in small teleosts and mammals

### The structural and developmental characteristics of muscle

Using zebrafish to study sarcopenia can greatly benefit from the extensive basic developmental research conducted on fish myogenesis and regeneration. Since the maintenance of skeletal muscle mass relies on a dynamic balance between degradation and regeneration, comprehensively understanding these processes is also imperative for the development of potential treatments.

The overall course of somite development in zebrafish is similar to that of amphibians, birds and mammals (Kimmel et al., 1995). However, it must be recognized that both conserved and divergent aspects of embryonic myogenesis, muscle anatomy and growth exist between teleosts and mammals (Keenan and Currie, 2019). A substantial portion of the zebrafish body is composed of skeletal muscle, remarkably similar to human muscle at both cellular and molecular levels, myofiber and sarcomere ultrastructural levels, and contractile properties. Zebrafish muscle development is much faster than that of mammals, with skeletal muscles spontaneously starting to contract at 17 h post-fertilization (hpf). By 24 hpf, all segmental blocks of muscle, or myotomes (Box 1), are present, partitioned by tendon-like junctions, enabling the embryo to coil and even respond to touch. The muscle becomes fully differentiated by 48 hpf and the larva is freely swimming by 96 hpf (Baxter and Bryson-Richardson, 2018; Stickney et al., 2000; Maves, 2014).

Zebrafish skeletal muscle is segmented into repeated chevron-shaped myotomes, composed of slow and fast muscle (Box 1) fibers organized along the head-to-tail axis (Lin, 2012; Baxter and Bryson-Richardson, 2018). The partition between each pair of adjacent somites is termed the vertical myoseptum, and the one between the dorsal and ventral halves is termed the horizontal myoseptum. These myosepta are where differentiated muscle fibers anchor, and function in force transmission during movement. The extracellular-matrix-rich junctional areas between muscle fibers and the myosepta are equivalent to the myotendinous junctions in mammals (Chen and Galloway, 2017). Myosepta are considered analogous to mammalian tendons, based on their structural, developmental and molecular similarities (Bricard et al., 2014). Additionally, the mechanical function of zebrafish myosepta declines significantly with age, as observed in mammalian tendons, confirming the utility of zebrafish for the study of tendon biomechanics and aging (Shah et al., 2015).

Vertebrate skeletal muscle is a heterogeneous tissue, composed of individual muscle fibers that are diverse in size, shape, contractile performances and protein content, so they can fulfill diverse functional needs. In both embryo and adult zebrafish, slow and fast myofibers of the trunk are spatially separated, in contrast to amniotes such as mammals, in which different fiber types are intermixed within muscle bundles (Keenan and Currie, 2019; Bassett and Currie, 2004; Lin, 2012). In the adult zebrafish, slow fibers are positioned in a wedge-shaped triangle on the lateral surface of the myotome, whereas fast fibers, which constitute most of the trunk musculature, are located in the deep portion of the myotome, attached at an oblique angle. Additionally, as in human muscle, zebrafish also possess an intermediate population of fibers, only a few cells thick, located between the slow and fast myofibers. Intermediate fibers maintain the contractile properties of fast fibers with a resistance to fatigue typical of slow fibers (Talbot and Maves, 2016). Furthermore, slow fibers in zebrafish are mononucleated, whereas fast fibers are multinucleated, unlike in mammals (Biressi et al., 2007).

Although muscle development in mammals and zebrafish is similar, there is an important functional difference between the two: the zebrafish external cell layer, which contributes to muscle growth, does not give rise to primary myogenesis, and, most importantly, it persists beyond embryonic myogenesis and continuously contributes new muscle fibers. This trait is in contrast to the amniotic dermomyotome, which is a transient embryonic structure (Stellabotte and Devoto, 2007; Delfini et al., 2009). Adult zebrafish skeletal muscle contains Pax7 (also known as Pax7a)-expressing cells interspersed throughout most of the myotome, which are functionally equivalent to mammalian satellite cells (Hollway et al., 2007; Gurevich et al., 2016). These satellite-like cells are enriched in slow muscle near the myosepta and are a source of new myofibers during adult zebrafish skeletal muscle repair and regeneration (Gurevich et al., 2016; Berberoglu et al., 2017).

### Mechanisms of muscle growth

Skeletal muscles grow through two types of mechanisms: hypertrophy, which is an increase in the diameter or length of existing muscle fibers, and hyperplasia, the addition of new muscle fibers, or a combination of the two (Hasumura and Meguro, 2016). Many amniotes, including mice and humans, finish the production of their full set of skeletal muscle fibers soon after birth, when hyperplastic growth completely ceases and they almost fully depend on hypertrophy for muscle growth (White et al., 2010). Postnatal production of new muscle fibers in most mammals is observed only in regenerating injured muscle, resulting in limited growth restricted by a determinate body size (Dhawan and Rando, 2005). In many large teleosts, skeletal muscles grow through both hyperplasia and hypertrophy throughout their entire lifespan (Mommsen, 2001), alongside a continuous increase in their body size. Small fish such as zebrafish, medaka and killifish are characterized by a lifelong muscle hyperplasia that becomes very limited after reaching the juvenile stage (Kishi, 2004; Genade et al., 2005; Gerhard, 2007). During the juvenile and adult stages, zebrafish go through age-related decrease in growth rate, and muscle growth primarily depends on hypertrophy (Bryson-Richardson and Currie, 2008; Patterson et al., 2008). Understanding the natural mechanisms responsible for lifelong hyperplastic muscle growth in teleosts presents a great potential for deciphering age-related muscular disorders in mammals, such as sarcopenia.

### Energy metabolism and intramuscular fat

Besides muscle tissue, the muscle contains three other major network components: connective (including adipose), vascular and nervous tissue. Additionally, the biochemical composition of skeletal muscle mostly constitutes of a different proportion of organic and inorganic substances, such as 75% water, 20% protein, 1-10% fat and 1% glycogen. Likewise, 90% of muscle material is occupied by fast (white), slow (red) and intermediate (pink) muscle fibers and the remaining 10% is intramuscular connective tissue. It is clear that the optimal concentration of each chemical, biological and biochemical component of muscle tissue is essential to provide muscle fiber organization and maintain its functional stability throughout an organism's lifetime (Listrat et al., 2016).

Intramuscular fat is observed in humans and small fish models alike. This intramuscular fat is mainly made up of saturated, unsaturated and storage lipids. Studies are suggesting that muscle-fiber-type-specific lipid metabolism could be an emerging research topic, aiming to investigate muscle structural and morphological information for developing new drug target molecules for treating muscle disorders like cachexia and sarcopenia (Capel et al., 2019).

Genetic, epigenetic and environmental factors significantly alter structural, biochemical and functional properties, indicating that accumulation of intramuscular fat may cause damage and dysfunction of a muscle network system (Akazawa et al., 2018). Nevertheless, it is not yet clear whether intramuscular fat decreases muscle fiber number, size and muscle volume, thus leading to muscle wasting in sarcopenia.

### Muscle regeneration in teleosts

Muscle damage is caused both by biological factors, such as elevated inflammation markers, cytokines (e.g. IL-10 and TNF-α), and by physical factors, such as trauma and injury to the muscle tissue. In the muscle regeneration cascade, pro-inflammatory markers play a critical role in controlling active immune response to maintain routine muscle regeneration functions. Similarly, many reports are suggesting that alteration of metabolic regulatory factors associated with lipid metabolism promotes muscle injury, but the mechanism of muscle healing/regeneration remains to be elucidated (Markworth et al., 2016).

Muscle regeneration is mainly carried out by its tissue-specific resident stem cells. Hence, the balance between muscle stem cells (MuSCs) and other MuSC-related cofactors is essential to activate muscle regeneration. MuSCs dynamics are largely controlled by many intrinsic and extrinsic factors. To date, it is unclear whether impaired muscle regeneration and loss of muscle-fiber-type-specific myogenic markers, like Pax3 and Pax7, would lead to sarcopenia. Another unknown is whether the inactivation of muscle quiescent cells at muscle-tendon junctions, which causes a delay in muscle fascicle fusion for regeneration, is due to aging or to accumulating lipids (Yin et al., 2013).

Live imaging studies in zebrafish (e.g. Gurevich et al., 2016) showed that quiescent satellite cells, activated upon injury, undergo asymmetric division, which results in self-renewing or proliferating cells. Proliferative cells undergo myogenesis to generate *de novo* immature fibers. This *in vivo* finding – confirming the initial *in vitro* only results – resolved a long-term debate surrounding the existence of this stem cell self-renewal and muscle repair mechanism *in vivo*. Improved understanding of this mechanism could help identify therapeutic windows for muscle-wasting conditions such as sarcopenia.

## Studies of muscle phenotypes in zebrafish models

Human and zebrafish muscle-related genes have broad functional conservation in muscle growth and development, implying that zebrafish is an excellent model to study human diseases such as myopathies and muscular dystrophies (Gupta et al., 2013). To study human disease-causing variants, researchers have established genetic zebrafish models by utilizing random chemical mutagenesis [using N-ethyl-N-nitrosourea (Henke et al., 2017)] and cutting-edge targeted genomic mutagenesis technologies such as zinc finger nucleases (ZFNs), transcription activator-like effector nucleases [TALENs (Radev et al., 2015)] and clustered regularly interspaced short palindromic repeats [CRISPR/Cas9 (Shi et al., 2018)] technology. The establishment of a zebrafish genetic model was primarily based on the molecular and functional characterization of genetic variants presumably associated with muscle phenotypes (Berger et al., 2011). Zebrafish models were explored by overexpression versus knockdown experiments. Existing zebrafish models are promising for studying distinct biological processes, signaling pathway regulation, ribosome biosynthesis, skeletal muscle growth and regeneration (Bennett et al., 2018). As a result, there are several zebrafish genetic models presenting muscle phenotypes that are available for the pharmaceutical industry for the discovery of effective treatments for human myopathies, muscular dystrophies (Kawahara et al., 2011; Horstick et al., 2013) and potentially other muscle-wasting conditions.

We summarize notable zebrafish studies in Table 1. For example, the *sapje* zebrafish strain carries a mutation in the dystrophin (*dmd*) gene that is responsible for altered protein-coding sequences with impaired cellular functions, thus causing progressive muscle wasting and loss of muscle integrity, similar to the phenotype of human *DMD* mutations in Duchenne muscular dystrophy (Bassett et al., 2003). Similarly, junction receptors are critical for maintaining muscle and tendon homeostasis by interacting with muscle fusion proteins at the cellular level. A mutation in the human *MYBPC1* gene leads to a progressive development of distal arthrogryposis (congenital contracture syndrome). Consequently, zebrafish *mybpc1* knockdown demonstrated abnormal developmental phenotypes such as a decrease in embryo hatching, impaired motor function and reduced number of sarcomeres (Ha et al., 2013).

**Table 1.**
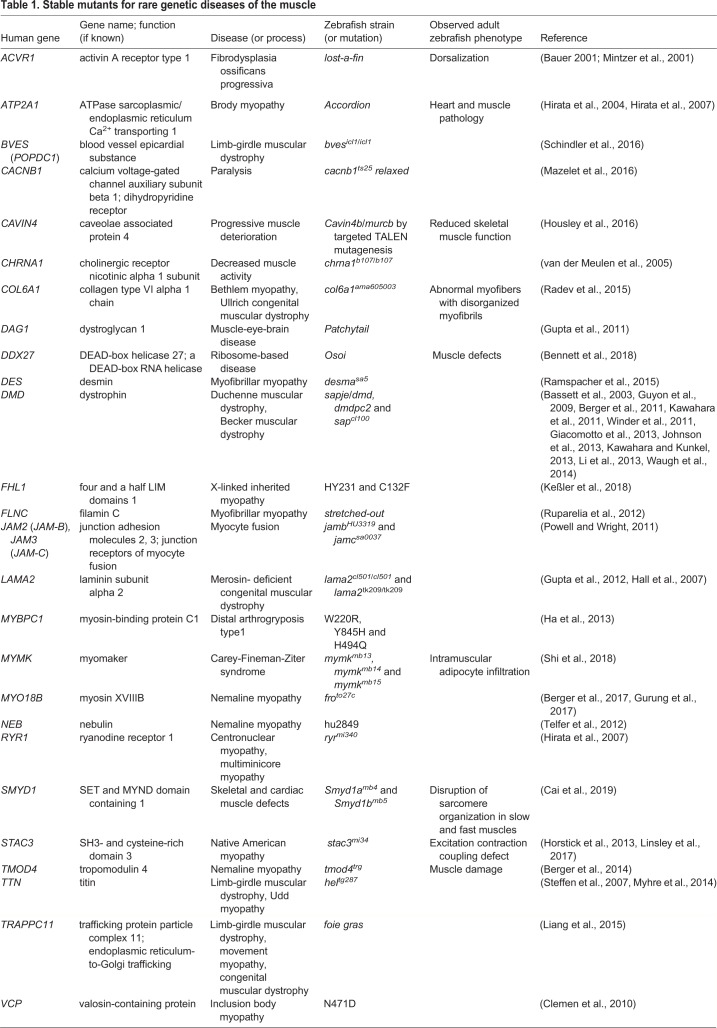
**Stable mutants for rare genetic diseases of the muscle**

In human infants, the N471 mutation in the nebulin (*NEB*) gene causes muscle weakness and decreased motor functions. The *neb* zebrafish genetic model that harbors the same mutation manifests similar histological features to humans, including decreased force generation and alterations in the muscle filament organization (Telfer et al., 2012). In addition, mutation in the human collagen type VI gene (*COL6A1*^ama605003^) causes Bethlem myopathy (Box 1) and Ullrich muscular dystrophy (Box 1). The corresponding mutation in zebrafish was the first model used to show collagen-associated muscle myopathy phenotypes, such as muscle fiber disorganization and their folding kinetics (Radev et al., 2015).

Proteostasis is one of the essential cellular biological processes that controls protein synthesis, degradation, localization and proper folding in subcellular compartments. Muscle integrity is retained by structural and functional proteins, such as actin and myosin, and sarcomere components, such as myofibrils and others (Guyon et al., 2009). Many genetic and environmental, especially stress-related, factors are responsible for increased protein degradation by altering protein homeostasis, which can cause myopathies and other proteostasis-related disorders like Parkinson's and Alzheimer's disease. Ultimately, maintaining protein stability and synthesis, an area of intense research, may reverse loss of muscle mass and declined muscle function.

With an intent to treat muscle wasting diseases like cachexia and sarcopenia, researchers extensively study the functional and structural involvement of myostatin (encoded by the *MSTN* gene). Myostatin is a negative regulator of skeletal muscle growth and development and the most explored member of the TGF-β (also known as TGFB1) superfamily (Bodnár et al., 2014). It has more than one biological function in cells and tissues, where it interferes with the protein synthesis machinery and, in particular, degrades muscle protein contractile functions (Rasal et al., 2016). All mutants – from mammals to small fish – exhibit dual muscular phenotypes, like hypertrophy and hyperplasia, caused by *MSTN* inactivation. *Mstn* knockout has resulted in muscle hyperplasia and muscle hypertrophy phenotypes in yellow catfish, medaka and rainbow trout (Rasal et al., 2016).

Myostatin gene functions are tightly controlled by several growth-promoting factors and post-translational modification of signaling cascades that activate myocyte proliferation and differentiation. In addition, myostatin is negatively regulated by many cell/tissue-specific proteins, lipids, enzymes and mutations in protein-regulatory domains. So far, several studies have successfully established myostatin models to investigate the dual muscular phenotypes and translate the novel biological findings into drug discovery (Saitoh et al., 2017). However, negative regulation of myostatin is unexplored in zebrafish skeletal muscle tissue. Unlike in humans and rodents, zebrafish possess two myostatin genes due to the duplication of their genome (discussed in more detail below).

Predominantly, the zebrafish genetic models for myopathies and muscular dystrophies are functionally and molecularly characterized via muscle-associated phenotypes such as locomotion, fatigue (muscle weakness), muscle wasting, motor function, muscle fiber development and sarcomere organization. All these phenotypes are usually assessed at embryonic and larval stages. It would be imperative to assess similar muscle phenotypes in adult fish to better understand sarcopenia (Berger et al., 2011; Telfer et al., 2012; Radev et al., 2015). Moreover, currently, no zebrafish genetic models are available to study age-related loss of muscle mass and impaired muscle function. Given the many advantages of zebrafish physiology, further investigation is needed to develop adult zebrafish models of muscle wasting for finding out novel, easily accessible drug targets and specific biomarkers for sarcopenia.

## Genomic structure: the teleost genome duplication and its consequences for disease modeling

The zebrafish genome is ∼1.4 billion base pairs in length, less than half the size of the human genome. Zebrafish possess 25,591 protein-coding genes (GRCz11 assembly, 2017; https://www.ncbi.nlm.nih.gov/assembly/GCF_000002035.6/), more than any previously sequenced vertebrate, probably as a consequence of whole-genome duplication (WGD). Several rounds of WGD have occurred early in the evolution of vertebrates, although there has been ongoing debate on the timing of these events and how many occurred (Sacerdot et al., 2018; Smith et al., 2018). Later, ∼226-350 million years ago, the ancestor of most teleost fishes, including zebrafish, underwent an additional round of WGD, termed the teleost genome duplication (Braasch et al., 2016; Hoegg et al., 2007). Approximately 80% of the zebrafish's duplicated genes were secondarily lost, yet some developed new functions or diverged into subfunctions. The zebrafish genome, therefore, includes many gene duplicates (termed ohnologs; Box 1), which are not represented in the human genome. This distinguishing aspect may be seen as a disadvantage, as it can prevent direct extrapolation to mammals, but sometimes it can be beneficial as it allows a more detailed dissection of an ancestral gene functionality (Amores et al., 2011; Postlethwait et al., 2004). For example, when a mutation of the mammalian ortholog (Box 1) causes embryonic lethality, mutating only one of the zebrafish paralogs at a time may give rise to less-severe phenotypes and viable embryos. For instance, the human troponin C1 gene (*TNNC1*) is expressed in both cardiac and skeletal muscle and is associated with certain forms of familial cardiomyopathy (Hoffmann et al., 2001). The zebrafish genome contains two paralogs of this gene (*tnnc1a*, *tnnc1b*) (Genge et al., 2013). This tandem gene duplication resulted in subfunctionalization and tissue-specific expression of each paralog: *tnnc1a* is mainly expressed in the heart, whereas *tnnc1b* is expressed in skeletal muscle (Sogah et al., 2010). Such separation allows analyses that would not be possible in mutant mammals, enhancing our knowledge of the functional link between human mutations and diseases.

### High conservation in coding DNA

Although teleosts diverged from lineages leading to humans more than 400 million years ago, a comparison of the zebrafish and human protein-coding genes reveals high genetic similarity, with over 70% of human genes having at least one functional zebrafish ortholog. Furthermore, 82% of all potential disease-related genes listed in the Online Mendelian Inheritance in Man (OMIM) database have at least one ortholog in zebrafish (Santoriello and Zon, 2012; Howe et al., 2013), enabling us to study their specific roles in this model system. Steffen et al. (2007) searched the zebrafish genome for 29 human muscular dystrophy genes and identified orthologs for 28 of them. In a more recent systematic literature review, Baxter and Bryson-Richardson (2018) noted that zebrafish models currently exist for 75 of 121 (62%) of all known skeletal myopathy-associated genes. Most of these genes also show conserved syntenic relationships with humans, reflecting important functional and regulatory relationships between these orthologous genes (Irimia et al., 2012).

Despite the teleost genome duplication, zebrafish chromosomes are mosaically orthologous to several human chromosomes (Postlethwait et al., 2000). The conservation of synteny is thought to represent adjacent genes that remain close to each other because they depend on each other and share coordinated transcriptional regulation, common cis-regulatory elements and gene expression, which is why they remain spatially linked through evolution.

### Divergence in non-coding DNA

Although protein-coding DNA is highly conserved among vertebrates, it covers only ∼1.5% of the human genome, while the rest is non-coding. The massive amount of data produced in recent years have confirmed that the majority of the evolutionarily constrained non-coding DNA (∼8% of the human genome) serves as protein-binding sites (Rands et al., 2014; Skipper et al., 2015). These conserved non-coding elements (CNEs) are usually located near genes, and are involved in the regulation of transcription and development. Functional assays of hundreds of CNEs have shown that they frequently function as cis-acting regulators (or enhancers), directing tissue-specific expression in early developmental stages (Lee et al., 2011; Pennacchio et al., 2006). Whole-genome comparative analysis of CNEs from teleosts and other vertebrates reveals that ∼80% of them have been lost in teleosts or diverged beyond recognition, apparently because of the accelerated teleost sequence evolutionary rate compared to other jawed vertebrates (reviewed in Lee et al., 2011; Ravi and Venkatesh, 2018). Nevertheless, some human CNEs can be directly connected to zebrafish. For example, Hiller et al. (2013) identified 54,533 zebrafish CNEs, of which ∼12,000 (22%) are conserved to human or mouse. More recently, Braasch et al. (2016) identified over 34,000 human CNEs conserved in zebrafish based on a direct whole-genome alignment. As more teleost fish genomes are being sequenced, Clément et al. (2020) were able to identify ∼1,300,000 human and ∼111,000 zebrafish CNEs relying on evolutionary sequence conservation, then to link them to target genes based on a synteny conservation score. As we pointed out in the GWAS section above, it is well recognized that ∼90% of disease-associated SNPs are mapped to non-coding intergenic or intronic regions and are not positioned within protein-coding genes, and therefore are likely to influence gene regulation (Buniello et al., 2019; Giral et al., 2018). In order to validate the function and connection to specific GWAS SNPs, researchers can use the CRISPR/Cas9 system to knock out or mutate conserved CNEs and study their functional role in the associated disease or trait (for example, see Madelaine et al., 2018).

Vertebrate genomes contain numerous non-coding RNA genes (ncRNAs), broadly classified according to the size of their mature transcripts as either small (<200 nucleotides; sRNAs) or long (>200 nucleotides; lncRNAs). Unlike the low retention rate for duplicated protein-coding genes, zebrafish microRNA (miRNA) ohnologs were conserved at a considerably higher rate of 39% (Braasch et al., 2016), consistent with the hypothesis that miRNA genes are likely to be retained after a duplication, on account of their integration in multiple gene regulatory systems (Loh et al., 2011). miRNAs regulate their mRNA targets post-transcriptionally, while lncRNAs affect the expression of neighboring genes by different mechanisms (St. Laurent et al., 2015) and play an imperative regulatory role in different biological processes, from development and differentiation to tumor progression in numerous metazoans (Mercer et al., 2009; Perry and Ulitsky, 2016). Unlike protein-coding genes, it is very challenging to directly annotate the functions of lncRNAs by sequence analysis due to the lack of information regarding their functional and structural domains. A recent large-scale computational analysis integrating various public zebrafish RNA sequencing datasets has discovered and annotated 13,604 zebrafish lncRNA genes (Chen et al., 2018), including 1890 transcripts with putative mammalian orthologs, accounting for ∼9% of total zebrafish lncRNAs. This result is in line with the low level of lncRNA conservation documented in earlier studies (Ulitsky et al., 2011). In some cases, however, although the human and fish lncRNAs do not align with each other in whole-genome alignments, the human orthologs can phenotypically rescue zebrafish lncRNA knockdown, e.g. the Cyrano lncRNA, which is crucial for proper morphogenesis and neurogenesis in zebrafish embryonic development (Ulitsky et al., 2011). These observations indicate that functionality is conserved across species, and therefore the discovery of additional less-conserved homologous lncRNAs may require the integration of syntenic data (Necsulea et al., 2014) for proper understanding of non-coding genome regulatory mechanisms.

Overall, improved understanding of both the coding and non-coding genome in teleosts will help researchers to generate relevant models for functional assessment of many GWAS-identified variants, particularly those in the non-coding genome, associated with complex conditions, such as sarcopenia.

## Conclusions

**Fig. 2.**
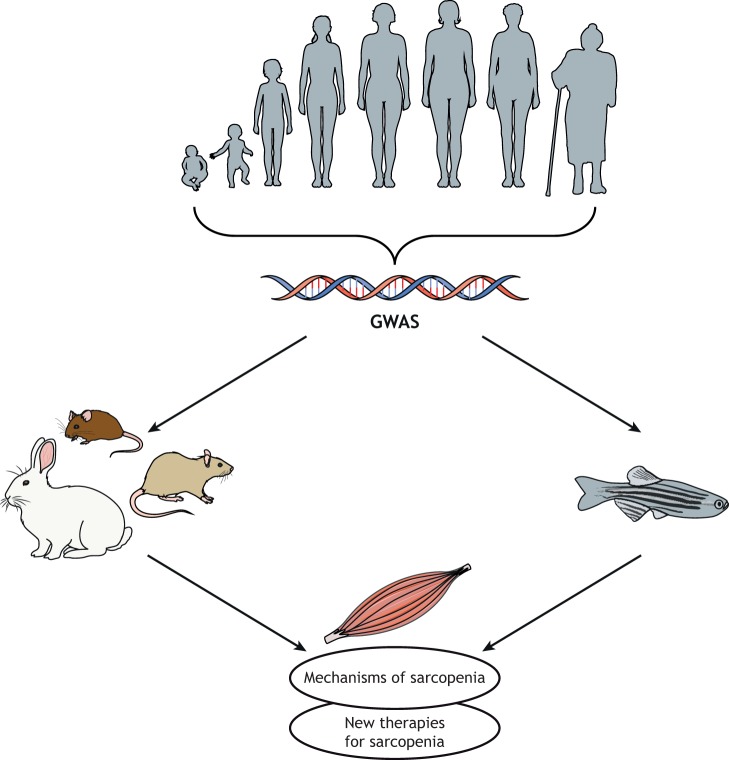
**Using model systems to understand and treat sarcopenia.** Aging is associated with loss of muscle mass and function, which are highly heritable traits. Combining GWAS with both traditional (rodent and rabbit) and teleost fish (mainly zebrafish) models allows researchers to improve our understanding of the mechanisms of muscle wasting and to search for new therapies. GWAS, genome-wide association studies.

Sarcopenia is an aging disease characterized both by loss of muscle mass and altered muscle function. Notably, the major sarcopenic phenotypes – muscle mass and muscle strength – are highly heritable. Nevertheless, our knowledge of genetic pathways responsible for regulating muscle-related traits is still far from complete. Owing to the resolution of GWAS, the causative gene at a particular locus often remains unidentified. Thus, to fully harness the power of this genetic approach, the community will need to embrace extensive and often costly functional testing. As of now, many studies are focusing on understanding the mechanisms related to muscle pathophysiology using rodent models. Yet, many researchers have developed reliable zebrafish models to understand muscle degeneration and its pathophysiology (Fig. 2).

Despite some anatomical differences, zebrafish muscle fibers are both functionally and ultrastructurally very similar to their amniote counterparts. Phenotypes of teleost muscle structure are well understood; muscle strength less so. Investigating abnormal myogenesis and its molecular mechanisms may help understand why synthesis of new muscle fibers, which is indispensable for (re)building healthy muscle tissue, is delayed in muscle-wasting conditions.

In this Review, we have outlined the challenges in interpreting zebrafish mutant phenotypes and translating these findings to human skeletal muscle disease. Other small fishes, like medaka and killifish, are also indispensable for skeletal muscle genomic studies, mostly due to the economy of scale achieved by raising a large number of genetically identical organisms. This knowledge is also important for aquaculture, where genetic and environmental factors can be modulated to achieve fish production efficiency. We thus believe that this Review article provides directions to leverage the large body of tools developed in small teleost for the needs of genomic exploration in sarcopenia and other age-related muscle conditions. Ultimately, the established zebrafish genetic models would provide added information for pharmacological intervention, and for implementing effective treatment for muscle disorders such as cachexia and sarcopenia.
